# High-Density Lipoprotein-Associated Paraoxonase-1 (PON-1) and Scavenger Receptor Class B Type 1 (SRB-1) in Coronary Artery Disease: Correlation with Disease Severity

**DOI:** 10.3390/jcm13185480

**Published:** 2024-09-15

**Authors:** Manish Kumar, Wahid Ali, Kusum Yadav, Swati Kaumri, Sridhar Mishra, Paolo Nardi, Ferdinando Iellamo, Sergio Bernardini, Akshyaya Pradhan, Marco Alfonso Perrone

**Affiliations:** 1 Department of Pathology, King George Medical University, Lucknow 226003, Uttar Pradesh, India; mankrshukla95@gmail.com (M.K.); drswati.rmlims@gmail.com (S.K.); 2 Department of Pathology, Dr. Ram Manohar Lohia Institute of Medical Sciences, Lucknow 226003, Uttar Pradesh, India; kusummedico9453@gmail.com (K.Y.); shridhar.mishra17@gmail.com (S.M.); 3 Department of Surgical Sciences, University of Rome Tor Vergata, 00133 Rome, Italy; paolo.nardi@uniroma2.it; 4 Division of Cardiology and CardioLab, Department of Clinical Sciences and Translational Medicine, University of Rome Tor Vergata, 00133 Rome, Italy; iellamo@uniroma2.it (F.I.); marco.perrone@uniroma2.it (M.A.P.); 5 Department of Experimental Medicine, University of Rome Tor Vergata, 00133 Rome, Italy; bernardini@med.uniroma2.it; 6 Lari Cardiology Center, King George Medical University, Lucknow 226003, Uttar Pradesh, India; akshyaya33@gmail.com

**Keywords:** coronary artery disease, atherosclerosis, Paraoxonase-1, Scavenger Receptor Class B Type 1, high-density lipoprotein

## Abstract

**Background:** Coronary artery disease (CAD) is the leading cause of death worldwide. High-Density lipoprotein (HDL) is a well-established marker associated with CAD. The current research goes beyond the conventional HDL-C measurement in previous studies and dives into the functional intricacies of HDL. By understanding how HDL works, rather than just how much of it exists, we can better tailor diagnostic and therapeutic strategies for CAD and related conditions. Hence, the current study quantifies the serum levels of two novel HDL-associated markers, Paraoxonase-1 (PON-1) and Scavenger Receptor Class B Type 1 (SRB-1), in CAD cases vs. controls. **Methods:** A total of 92 subjects, including 69 CAD and 23 healthy controls, were included, based on the prevalence of the disease. Further, based on the severity of the disease, CAD cases were subcategorized as CAD-I, -II, and -III. Serum PON-1 and SRB-1 levels were measured and compared between patient and control groups. **Results:** The levels of PON-1 and SRB-1 (32.6 ng/mL and 12.49 ng/mL) were significantly lower in CAD patients vs. the healthy control, at 60.36 ng/mL and 15.85 ng/mL, respectively (*p* < 0.000). A further intergroup comparison showed a statistically significant difference between the CAT-I and -III for PON-1 (*p* < 0.025), the CAT-I and -III, and CAT-II and -III for SRB-1 (*p* < 0.000). The receiver operating characteristics (ROC) curve showed cutoff values of 48.20 ng/mL and 14.90 ng/mL for PON-1 and SRB-1. **Conclusions:** The current study found that serum levels of HDL-associated PON-1 and SRB-1 are significantly lower in CAD cases, and were also inversely related to the increasing severity of coronary artery disease. This inference implies that serum PON-1 and SRB-1 could be used as non-invasive tools for the identification of coronary atherosclerosis and risk assessment in CAD cases.

## 1. Introduction

Cardiovascular disease (CVD) is the leading cause of death worldwide. Approximately 17.7 million people worldwide suffer from various types of cardiovascular diseases each year [1]. Ischemic heart disease (IHD) and stroke are the leading causes of cardiovascular disease-related deaths in India (~83%) [2]. The protective response of High-Density Lipoprotein (HDL), and the atherogenic effect of Low-Density Lipoprotein (LDL), are well-established risk factors for coronary artery disease (CAD) development. HDL has numerous effects on the function and integrity of endothelial cells, e.g., endothelial anti- and prothrombotic activity, modulation of nitric oxide (NO) production, tissue repair, apoptosis, and adhesion of molecules. The aforementioned functions are mediated by Scavenger Receptor Class B Type 1 (SRB-1) and S1P3 receptor-dependent signaling pathways [3,4].

HDL stimulates the production of NO via the endothelial nitric oxide synthase (eNOS) enzyme, and it binds via the SRB-1 receptor. NO plays a crucial role in the protective response, including vasodilation, anti-inflammatory, and anti-adhesion effects [5]. Much emphasis has been placed on the HDL value, not only on the numerical values, but also on the faulty HDL’s functioning; for example, B. Functional HDL deficiency is the underlying cause of the development of atherosclerosis [5]. Experiments with knockout mice have shown that the antioxidant property of HDL is largely due to the Paraoxonase-1 (PON-1) enzyme that is found on it [6,7]. PON-1 is an enzyme primarily associated with HDL particles. It has antioxidant properties and plays a role in protecting against oxidative stress. PON-1 enhances cholesterol efflux from macrophages in atherosclerotic plaques. This process helps prevent plaque from progressing and breaking down. Inflammation is a major trigger of atherosclerosis, and PON-1 has been found to reduce inflammation by inhibiting the oxidation of Low-Density Lipoprotein (LDL) cholesterol. In addition to this, PON-1 can detoxify certain organophosphate compounds, which may indirectly affect CAD risk [8]. Low serum PON-1 activity is significantly reduced in patients with myocardial infarction (MI), which may be the reason for the development of CAD [8]. Research has shown that the genetic polymorphism in PON-1 leads to a reduction in LDL peroxidation prevention activity and the development of atherosclerosis [8]. The potential assaying of PON-1 may be more effective than conventional serum HDL levels in predicting atherosclerosis in patients with type II diabetes mellitus [9].

SRB-1 is an important component of reverse cholesterol transport because it binds to HDL and facilitates the selective transfer of lipids [10,11]. It is critical for cholesterol sensing and facilitates bidirectional cellular cholesterol flow [11]. The role of SRB-1 in knockout mice was investigated, and it was found that knockout mice lacking SRB-1 reduce cholesterol reverse transport and increase the risk of developing atherosclerosis [11,12]. On the other hand, SRB-1 overexpression in the liver reduces serum HDL levels and reduces the risk of atherosclerosis, suggesting that SRB-1 overexpression in the liver may promote cholesterol efflux through reverse cholesterol pathways [13]. It has been postulated that PON-1 and SRB-1 are part of the complex interplay between HDL particles, cholesterol metabolism, and inflammation. Various studies have shown that the balance between cholesterol uptake (via SRB-1) and cholesterol efflux (augmented by PON-1) is crucial. Dysregulation can lead to lipid accumulation and plaque formation. Variability in SRB-1 expression levels may impact cholesterol management and CAD risk. Understanding these mechanisms could guide therapeutic strategies. For example, increasing PON-1 activity or promoting SRB-1 expression could be beneficial. Genetic variations in PON-1 and SRB-1 may explain why some individuals are more susceptible to CAD, despite similar HDL-C levels [11,12,13].

The studies have reported inconsistencies in PON-1 polymorphism associations. While many studies have examined the association of PON-1 polymorphisms with CAD, results have not been consistent across the literature. Our study aims to provide clarity by analyzing the serum association of PON-1 levels with CAD severity. In addition, we address the role of SRB-1 in CAD. The existing literature provides some insights, but gaps remain. Our research aims to address these gaps and provide a comprehensive understanding of the relevance of SRB-1 to CAD pathophysiology. Hence, the current study was conducted to measure and correlate SRB-1 and PON-1 levels with the severity of coronary obstruction in patients with CAD, compared to the normal control group.

## 2. Material and Methods

Study participants were recruited between December 2021 and June 2022 from the Lari Cardiology Center, Department of Cardiology, King George Medical University, Lucknow, Uttar Pradesh, India in collaboration with the University of Rome Tor Vergata, Rome, Italy. Coronary angiograms were evaluated by a clinician who performed a visual assessment of luminal narrowing in multiple segments using the AHA/ACC classification of the coronary tree [14].

A total of 92 subjects were included in the study, 69 of whom had angiographically proven CAD and varying degrees of vessel stenosis severity. The diagnosis of coronary artery disease was made by a cardiologist based on the patient’s history, clinical examination, and relevant investigations, including angiography (whereby >50% stenosis in one or more coronary arteries was used as a diagnostic criterion). The degree of stenosis and severity of each case were evaluated by an experienced cardiologist and divided into three groups, namely Category 1, Category 2, and Category 3, based on the presence of angiographically proven single-, double-, and triple-vessel stenosis, respectively.

The healthy control group consisted of 23 subjects without detectable coronary stenosis or atherosclerotic vascular disease. Patients were interviewed to record their medical history and lifestyle habits. All patients who met the inclusion criteria were recruited for the study. Written informed consent was obtained from all patients. The research related to human use was conducted in accordance with all relevant national regulations and institutional guidelines, and in accordance with the principles of the Declaration of Helsinki. The study was approved by the authors’ institutional ethics committee of King George Medical University, Lucknow, before the start of the study. The exclusion criteria included cases with recent acute coronary syndrome (within three months), diabetes mellitus type I and type II, chronic kidney and liver diseases, active systemic infections, morbid obesity, and connective tissue diseases. All demographic data were collected from the OPD- and IPD-enrolled patients whose angiographic had already been performed.

### 2.1. Sample Collection and Serum Isolation

A total of 5.0 mL of peripheral blood was collected from 92 subjects in plain EDTA vials (NOVAC, POLYMED, Poly Medicure Ltd., New Delhi, India). The serum was separated by centrifugation at 1900× *g* for 10 min, followed by a 10 min high-speed centrifugation at 16,000× *g*, and stored at 80 °C until further processing.

### 2.2. Biochemical Examination

Biochemical parameters, including Total Cholesterol (TC), (mg/dL), Triglyceride (TG), (mg/dL), High-Density Lipoproteins (HDL-C) (mg/dL), Low-Density Lipoproteins (LDL-C) (mg/dL), and Very-Low-Density Lipoproteins (VLDL-C) (mg/dL), were recorded. All biochemical parameters were measured using a fully automated biochemical analyzer (ARCHITECT i2000SR, Abbott Diagnostics, Wiesbaden, Germany and Selectra ProXL, ELITech Group, Puteaux, France).

### 2.3. Enzyme-Linked Immunosorbent Assay for PON-1 and SRB-1

Serum PON-1 and SRB-1 levels were determined using an ELISA kit (Elabscience, Houston, TX, USA) based on the sandwich ELISA principle. After performing several steps, the stop solution was added, and the optical density (OD) was immediately measured spectrophotometrically at a wavelength of 450 nm ± 2 nm. Finally, based on this OD, the serum concentrations of PON-1 and SRB-1 were measured in ng/dL. To minimize intra-assay variability, the coefficient of variation (CV), which is the standard deviation divided by the mean (expressed as a percentage), was calculated. Intra-assay CV values less than 10% were found, and the results were considered reliable. Similar to the intra-assay CV, the inter-assay CV was calculated from the standard deviation and the mean of measurements over different assay runs, and it was less than 12%. All samples were routinely analyzed in triplicate by ELISA, and the results were averaged to minimize measurement errors and calculate the final concentration in the samples.

### 2.4. Statistical Analysis

Statistical analysis was performed using SPSS (Statistical Package for Social Sciences) Version 21.0 Statistical Analysis Software. Normally distributed continuous variables are presented as mean ± SD (standard deviation), and in number (%). The ANOVA test, followed by post hoc tests (Tukey-HSD), was used to compare the within-group and between-group variances. A receiver operating characteristic curve (ROC) was developed to evaluate the diagnostic performance of SRB-1 and PON-1. A 2 × 2 table was built to categorize tests as positive or negative based on individual cutoffs obtained from ROC curves. A *p* value < 0.05 (two-tailed) was considered significant.

## 3. Results

### 3.1. Demographic and Baseline Characteristics of the Patients and Controls

The demographic and clinical parameters of the patients and controls are presented in Table 1. When baseline characteristics were compared between the CAD cases and control groups, older age was observed in the CAD cases (*p* < 0.000). Male predominance was observed in the development of coronary heart disease (*p* < 0.000), along with a significantly higher number of subjects who were smokers, alcohol consumers, and non-vegetarians who developed coronary heart disease (*p* < 0.012, *p*< 0.052, and *p* < 0.048). Obesity, BMI, and hypertension showed no significant difference between the CAD cases and control groups, as shown in Table 1. The subject who attended the OPD, underwent angiography, and had no significant CAD was considered a normal control (non-significant CAD).

### 3.2. Lipid Parameters and PON-1 and SRB-1 in Cases and Controls

The lipid parameters and PON-1 and SRB-1 levels are shown in Table 2. Lower HDL levels were observed in CAD patients (44.76 ± 8.15 mg/dL) compared to controls (50.65 ± 12.43 mg/dL, *p* = 0.011), but no significant differences were found in LDL, VLDL, and TG levels, as shown in Table 2. A low level of PON-1 was observed in CAD cases (32.65 ± 14.67 ng/dL) compared to controls (60.36 ± 12.63 ng, *p* = 0.000). Furthermore, a significantly lower SRB-1 level was observed in CAD cases (12.49 ± 3.34 ng/dL) compared to controls (15.85 ± 2.76, *p* < 0.000), as presented in Table 2.

### 3.3. Intergroup Comparison of PON-1 and SRB-1 with Different Categories of CAD and Normal Control

**Figure 1:**
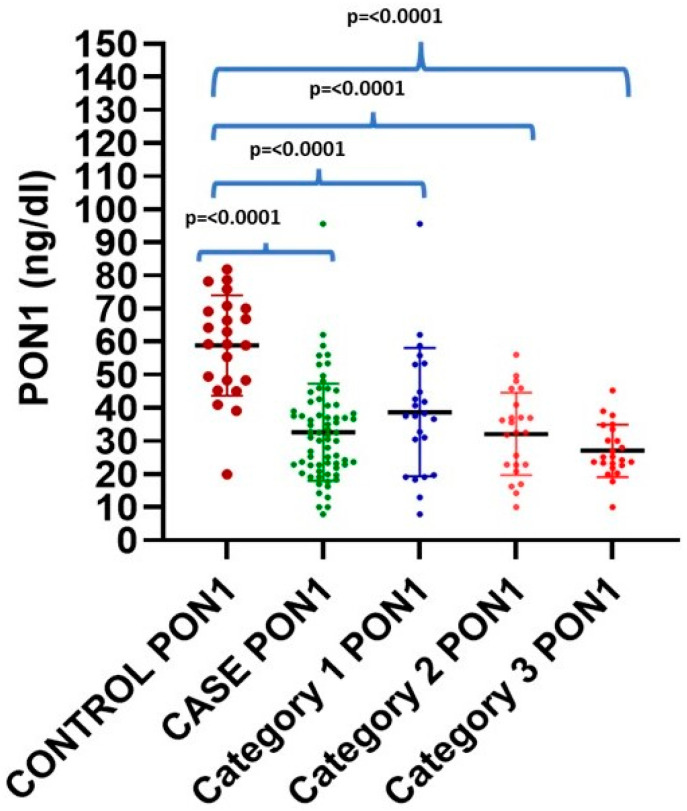
Scatter plot showing the level of PON-1 in cases and controls, as well as different categories of the CAD severity.

**Figure 2:**
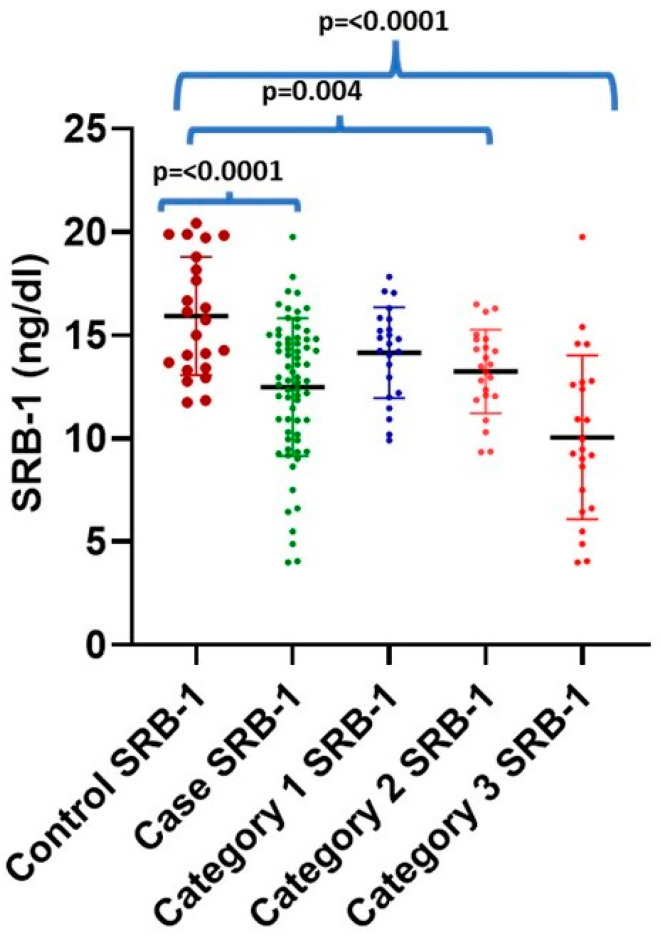
Scatter plot showing the level of SRB-1 in cases and controls, as well as different categories of the CAD severity.

PON-1 concentration was found to be inversely correlated with increasing severity of vascular obstruction, i.e., from a single-vessel obstruction to a triple-vessel obstruction (CAD-I to CAD-III), as shown in Table 3 and Figure 1. All categories (CAD-I, -II, and -III) show a statically significant difference compared to the controls (*p* < 0.000). Intergroup comparisons were performed in CAD patients, and a statistically significant difference was found between CAD-I (38.74 ± 19.41 ng/dL) and CAD-III (27.05 ± 7.86 ng/dL, *p* = 0.025). However, no statistically significant difference was found between CAD-I (38.74 ± 19.41 ng/dL) and CAD-II (13.25 ± 2.03 ng/dL), and between CAD-II and CAD-III (27.05 ± 7.86 ng/dL), as shown in Table 3. The maximum SRB-1 level was found in the control subject (20.43 ng/dL), while the minimum level (4.01 ng/mL) was found in CAD-III cases. It was observed that 17 out of 69 (24.63%) CAD patients had decreased SRB-1 levels, despite higher-than-normal HDL levels (mean 53.81, maximum 66.6 mg/dL) (mean 10.82, minimum 4.9 ng/dL). Similarly, SRB-1 levels were also found to be inversely related to increasing severity of vascular obstruction. CAD-II and CAD-III showed a statically significant difference compared to healthy controls (*p* = 0.014 and *p* = 0.000, respectively), as shown in Table 3 and Figure 2. An intergroup comparison was performed, showing a statistically significant difference between CAD-I (14.15 ± 2.20 ng/dL) and CAD-III (10.06 ± 3.98 ng/dL, *p* = 0.014), and CAD-II (13.25 ± 2.03 ng/dL) and CAD-III (10.06 ± 3.98 ng/dL, *p* = 0.001); however, no significant difference was observed between CAD-I (14.15 ± 2.20 ng/dL) and CAD-II (13.25 ± 2.03 ng/dL).

### 3.4. Association of PON-1 and SRB-1 Level with Demographic Characteristics

We have also analyzed the level of PON-1 and SRB-1 in CAD cases and controls with various other demographic and clinical parameters that have been established as risk factors for the development of coronary artery disease, including tobacco use, alcohol consumption, family history, obesity, and hypertension; these findings are presented in Table 4. The PON-1 level was significantly different, and higher, in cases that were presented with hypertension, compared to cases with no hypertension (*p* = 0.002). Furthermore, the PON-1 level was higher in cases with obesity compared to non-obese cases; however, the difference was not significant (*p* = 0.330).

### 3.5. Diagnostic Value of PON-1 and SRB-1 in Discriminating Cases from Controls

**Figure 3:**
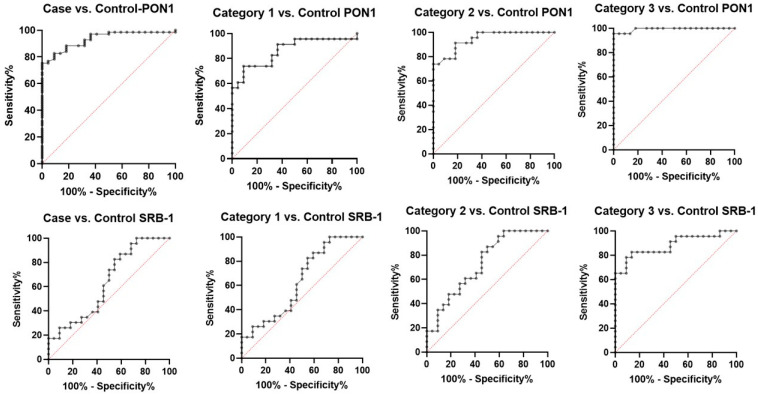
ROC curve showing the sensitivity and specificity of PON-1 and SRB-1 in cases and controls and with different categories of CAD severity.

To find out diagnostic accuracy (sensitivity and specificity) of markers (PON-1 and SRB-1) in predicting CAD, both of the markers were subjected to ROC curve analysis, and they were summarized in Table 5 and Figure 3. In the discrimination of CAD cases from controls, the area under the curve for PON-1 was 0.93. Furthermore, at a cutoff point of ≤39.10 ng/dL (PON-1), the sensitivity and specificity were 75.36% and 100.00, respectively. Moreover, we further calculated the diagnostic potential of PON-1 in the discrimination of CAD cases based on stenosis level, viz., CAD-I, CAD-II, and CAD-III, which were selected from the normal control. The AUC was highest for the discrimination of CAD-III from normal controls (AUC = 0.99), with a sensitivity and specificity of 95.65% and 100.00%, respectively. The AUCs for the discrimination of CAD-I and CAD-II were 0.86 and 0.94, respectively.

### 3.6. Diagnostic Value of SRB-1

When distinguishing CAD cases from controls, the area under the curve for SERB-1 was 0.75. Furthermore, the sensitivity and specificity at a cutoff value of 13.63 ng/dL (SRB-1) were 57.97% and 72.73%, respectively. In addition, we further calculated the diagnostic potential of SRB-1 in distinguishing CAD cases based on the degree of stenosis, viz., CAD-I, CAD-II, and CAD-III, from the normal control. The AUC was highest for distinguishing CAD-III from normal controls (AUC = 0.88), with a sensitivity and specificity of 78.26% and 90.91%, respectively. The AUCs for distinguishing CAD-I and CAD-II for SRB-1 were 0.62 and 0.72, respectively, as depicted in Table 5 and Figure 3.

## 4. Discussion

Asian Indians are more susceptible to developing coronary heart disease a decade earlier than the Caucasian population [15]. Furthermore, a recent study found that the risk of developing cardiovascular diseases is higher among North Indians compared to people from other parts of India. It is important to emphasize that the traditional risk factors have sometimes failed to fully justify the excess risk of coronary heart disease in Indians, raising the possibility of genetic susceptibility [16].

Our study showed that PON-1 levels decreased significantly in Category III (>90%), which included CAD cases with luminal stenosis, regardless of the number of affected coronary arteries. One study found a positive association between low PON-1 activity and the severity of coronary artery disease, particularly in smokers and diabetic patients [17]. We also found a significant association between PON-1 and hypertension in CAD cases. Therefore, serum PON-1 may have a prognostic value in determining the severity of CAD.

Oxidative damage plays an important role in chronic inflammatory diseases, such as CAD. PON-1 is atheroprotective due to its antioxidant and anti-inflammatory roles in the body [18,19]; indeed, it prevents LDL oxidation, thereby reducing the risk of atherosclerosis [20]. The diverse genetic variations in the PON-1 gene may influence its functional role, and thus its ability to protect against CAD. Our results showed that PON-1 levels were significantly lower in CAD patients compared to controls. Our data also showed that individuals with lower PON-1 levels had a higher risk of developing CAD. In addition, the serum PON-1 level was significantly lower in the CAD-III category compared to CAD-I and CAD-II, as well as the control group.

Several studies have been conducted to investigate the association between PON-1 polymorphism and serum PON-1 activity in CAD patients. Mackness MI et al. investigated that the PON-1 R allele polymorphism is associated with the reduced PON-1 concentration and an increased risk of developing coronary artery disease [6]. Fortunato G. et al. examined 310 middle-aged women for intimal thickness and PON-1 polymorphism, which are related to premature atherosclerosis, and found that PON-1 (LL/ML) seems to be positively correlated to intimal thickness and carotid atherosclerosis [21]. Coombes et al. examined PON-1 serum activity and concluded that an increase in PON-1 activity is associated with a reduction in the risk of atherosclerosis [22]. Bhaskar et al. conducted a cohort study in an Indian cardiovascular disease population with and without diabetes mellitus and found that the frequency of the PON-1-192RR genotype and R allele were both increased in CAD and T2DM patients, compared to controls [23].

PON-1 inhibits LDL-C oxidation and lipid peroxide accumulation in macrophages. Reduced uptake of oxidized LDL is likely to be mediated by PON-1 interaction with Scavenger Receptor Class B Type 1 (SRB-1) on the macrophage surface, leading to the suppression of macrophage proinflammatory responses [24]. PON-1 also reduces monocyte chemotaxis and adhesion to endothelial cells, thereby preventing endothelial damage and atherosclerosis [25]. Research also shows that incubation of PON-1 with HDL results in a reduction in the expression of intercellular adhesion molecule (ICAM)-1 on endothelial cells, which helps to reduce the progression of inflammation in the endothelium. PON-1 also protects against the proinflammatory effects of oxidized phospholipids and lipopolysaccharides. Furthermore, PON-1 reduces cholesterol biosynthesis by macrophages, and increases cholesterol efflux from LDL-C [26]. Some research has shown that in carriers of the RR genotype, PON-1 enzyme activity decreases, increasing oxidative stress and inflammation; furthermore, all of these events may be related to the occurrence of atherosclerosis and CAD [27].

The most important role of SRB-1 is the clearance of plasma cholesterol via the reverse cholesterol transport pathway (RCT), in which mature HDL particles transport cholesterol from peripheral tissues to the liver for excretion [28,29]. Lipid-poor HDL dissociates from SRB-1 and re-enters the circulation to continue participating in the RCT. Therefore, an efficient interaction between SRB-1 and HDL is required to maintain cholesterol homeostasis and protect against plaque accumulation that occurs in atherosclerotic cardiovascular disease (ASCVD). The importance of SRB-1 in atheroprotection is well-illustrated by in vivo studies [28,29,30,31]. When SRB-1 is genetically deleted in wild-type mice, HDL cholesterol (HDL-C) levels double, and atherosclerotic plaques rapidly develop [30,31]. SRB-1 deletion in atherosclerotic ApoE knockout mice results in myocardial infarction and early death [32]. Similarly, SRB-1 deletion in Low-Density Lipoprotein receptor (LDL) knockout mice fed a high-fat Western diet also shows early atherosclerosis [33,34]. Alternatively, SRB-1 overexpression in wild-type mice results in reduced HDL-C levels [35,36,37,38] and less atherosclerotic plaque formation [39]. In addition, all nine identified human variants of SRB-1 resulted in increased plasma HDL-C levels, with two variants (P376L and G319V-SRB-1) directly increasing the risk of ASCVD. Combining these clinical observations with mouse studies, the importance of SRB-1 in maintaining adequate HDL-C levels and mitigating ASCVD risk becomes increasingly clear [40,41].

Various animal model studies (knockout mice) have shown that mice lacking SRB-1 have an increased risk of developing atherosclerosis [11,12]. The present study also suggests that a similar mechanism is involved in the reverse cholesterol transport of patients with CAD, as there is a minimal SRB-1 level in patients with a triple-vessel stenosis. Huang L. et al. tested the hypothesis that SRB-1 is a receptor for HDL and LDL, and can thus promote atherosclerosis in the aorta and other atherogenic areas of mice [42]. They concluded that therapeutic interventions that inhibit the endothelial delivery of LDL into the arterial vials mediated by SRB-1 and DOCK 4 may be a potential remedy against the development of atherosclerosis and cardiovascular disease. Wang et al. investigated the function of a novel miR-24 on SRB-1 expression, HDL uptake, and lipid metabolism in different types of cells extracted from mice fed a high-fat diet, and found that miR-24 directly targets SRB-1 and decreases SRB-1 levels and HDL uptake [43]. Similarly, Shen et al. reviewed various research papers on the regulation and functional significance of SRB-1 in mediating cholesterol movement, and concluded that SRB-1 is a multiligand of membrane receptors that acts as a receptor for the selective delivery of HDL to cells, and that SRB-1 may serve as a potential marker for diagnostic and therapeutic purposes for the selective HDL uptake pathway in individuals with risk factors [44]. The current study also provides similar results and underlines the importance of SRB-1 in high-risk patients. In addition, Jiang C. et al. conducted an experiment on mice fed a high-fat and low-fat diet and concluded that SRB-1 plays an important role in adipogenesis in high-fat diet mice by regulating the increase in SRB-1 expression [45].

Zanoni et al. showed in their study that P376L carriers have an increased risk of CHD, despite their increased HDL level, and proved that steady-state concentrations of HDL-C do not provide causal protection against CHD and HDL function, and that cholesterol flux may be more important than the absolute values [40]. Zhang, Y. et al. examined the hepatic expression of SRB-1 and plasma HDL levels in mice and concluded that SRB-1-deficient mice have higher plasma HDL levels than mice with hepatic SRB-1 overexpression [13]. Zanoni et al. emphasized that people who are carriers of P376L have elevated HDL levels, but are at a higher risk of developing coronary artery disease [40]. This can be explained by defective expression of SRB-1 in the liver, which promotes the compensatory increase in absolute HDL levels; however, this increased HDL value does not protect against atherosclerosis, which is consistent with the experiment of Zhang, Y. et al. on mice [13]. Similar results were observed in our study. Our data showed that 17 of 69 (24.63%) CAD patients had varying degrees of coronary obstruction despite elevated HDL levels and reduced serum SRB-1 levels. The current study is an extension of previous animal model testing hypotheses, and demonstrates the association between atherogenesis and the role of SRB-1 in patients with CAD.

Our results support the inverse association between PON-1 and SRB-1 in patients with CAD. High-severity CAD cases have low PON-1 and SRB-1 levels compared to normal controls. Low levels of PON-1 and SRB-1 may be responsible for a change in the functionality of the PON-1 enzyme, leading to an increased risk of CAD. Furthermore, because CAD is a multifactorial disease due to the interaction of various environmental and genetic influences, there may also be other modulators that alter serum PON-1 levels in these individuals. Furthermore, PON-1 may have prognostic significance in severe coronary artery stenosis [46,47,48,49]. The limitation of our study was the relatively small sample size, representing only a fraction of patients with CAD admitted to a tertiary care hospital during the given period. However, our study shows the importance of additional risk factors, including hypertension, and valuable markers such as PON-1 and SRB-1 levels, which may be useful for predicting CAD in the Indian population. Prospective studies with a larger population are needed to gain a better understanding of the role of PON-1 in increased CAD susceptibility. PON-1 and SRB-1, as potential therapeutic candidates, are also interesting points for further study. Exploring PON-1 and SRB-1 in low-risk individuals could improve our understanding of early CVD mechanisms. Although there are challenges, this research can serve as a guide for personalized prevention strategies.

## 5. Conclusions

The present study was conducted with the primary aim of determining the serum levels of PON-1 and SRB-1, and their association with the severity of vessel stenosisin patients with CAD. Compared to healthy controls, significantly lower levels of PON-1 and SRB-1 were observed, with lower values in those angiographically diagnosed with the maximum severity of coronary stenosis. This knowledge can be used to assess the functional aspect of HDL, risk assessment of atherosclerosis in high-risk groups, and severity of coronary stenosis in patients with CAD. Based on the above data, a non-invasive, cost-effective serological diagnostic test can be developed. More studies will be needed to confirm these data. Although PON-1 and SRB-1 show promise as CAD biomarkers, their successful integration into routine practice requires overcoming technical, clinical, and logistical challenges. Clinicians, researchers, and policymakers must work together to manage this complexity and maximize the clinical impact of these biomarkers.

## Figures and Tables

**Table 1:** Basic and clinical characteristics of the study subjects.

Variables	Controls (*n* = 23)	CAD Cases (*n* = 69)	*p*-Value
Age (Years)	49.08 ± 14.09	57.55 ± 8.59	<0.000
Sex (Male/Female)	(14/9)	(65/4)	<0.000
Alcohol Consumer (N)	2 (8.6%)	25 (36.23%)	<0.012
Family History	2 (8.6%)	18 (20.0%)	0.486
Dietary Habits *	(13/10)	(23/46)	0.048
Nature of Work ^#^	(5/15/3)	(8/51/10)	0.480
Smoker (N)	6 (26%)	34 (49.27%)	<0.052
Hypertensive (N)	4 (17.39%)	24 (34.78%)	<0.116
BMI (kg/m^2)^	23.34 ± 3.43	24.22 ± 3.80	<0.325
Obesity ^$^	4 (17.3%)	21 (30.4%)	<0.223
Healthy Controls	23 (25%)	-	
Single-Vessel Stenosis (CAD-I)		23 (25%)	
Double-Vessel Stenosis (CAD-II)		23 (25%)	
Triple-Vessel Stenosis (CAD-III)		23 (25%	

* Dietary habits (Vegetarian/Non-Vegetarian); ^#^ Nature of Work (Hard/Moderate/Sedentary); ^$^ >30 BMI is considered as obese.

**Table 2:** Lipid parameters and PON-1 and SRB-1 levels in CAD cases and controls.

Variables	Controls (*n* = 23)	CAD Cases (*n* = 69)	*p* Value
Total Cholesterol (mg/dL)	142.4 ± 41.5	142.2 ± 40.1	0.983
High-Density Lipoprotein (HDL) (mg/dL)	50.65 ± 12.43	44.76 ± 8.15	0.011
Low-Density Lipoprotein (LDL) (mg/dL)	63.41 ± 31.96	67.29 ± 35.49	0.731
Very-Low-Density Lipoprotein (VLDL) (mg/dL)	33.03 ± 11.81	33.84 ± 17.90	0.840
Triglyceride (mg/dL)	166.67 ± 89.47	166.34 ± 88.07	0.942
PON-1 (ng/dL)	60.36 ± 12.63	32.65 ± 14.67	0.000
SRB-1 (ng/dL)	15.85 ± 2.76	12.49 ± 3.34	0.000
	**CAD-I**	**CAD-II**	**CAD-III**
SRB-1 (ng/dL)	38.74 ± 19.41	32.16 ± 12.45	27.05 ± 7.86
PON-1 (ng/dL)	14.15 ± 2.20	14.25 ± 2.02	10.06 ± 3.97

**Table 3:** Intergroup comparison for PON-1 and SRB-1 in controls and cases with different categories of disease severity.

Multiple Comparisons (Tukey-HSD)							
Dependent Variable	(I) GROUP	(J) GROUP	Mean Difference (I − J)	Std. Error	Sig. *	95% Confidence Interval	
						Lower Bound	Upper Bound
PON-1	CONTROL	CATEGORY-1	21.62 *	4.04	0.000	11.02	32.22
		CATEGORY-2	28.20 *	4.04	0.000	17.60	38.80
		CATEGORY-3	33.30 *	4.04	0.000	22.71	43.90
	CATEGORY-1	CATEGORY-2	6.58	4.04	0.369	−4.01	17.18
		CATEGORY-3	11.68 *	4.04	0.025	1.08	22.28
	CATEGORY-2	CATEGORY-1	−6.58	4.04	0.369	−17.18	4.01
		CATEGORY-3	5.10	4.04	0.590	−5.49	15.70
	CATEGORY-3	CATEGORY-1	−11.68 *	4.04	0.025	−22.28	−1.08
		CATEGORY-2	−5.10	4.04	0.590	−15.70	5.49
SRB-1	CONTROL	CATEGORY-1	1.69	0.83	0.190	−0.50	3.89
		CATEGORY-2	2.59 *	0.83	0.014	0.39	4.79
		CATEGORY-3	5.78 *	0.83	0.000	3.59	7.98
	CATEGORY-1	CATEGORY-2	0.90	0.83	0.707	−1.29	3.09
		CATEGORY-3	4.09 *	0.83	0.000	1.89	6.29
	CATEGORY-2	CATEGORY-1	0.90	0.83	0.707	−3.09	1.29
		CATEGORY-3	3.19 *	0.83	0.001	0.99	5.39
	CATEGORY-3	CATEGORY-1	−4.09 *	0.83	0.000	−6.29	−1.89
		CATEGORY-2	−3.19 *	0.83	0.001	−5.39	−0.99

* The mean difference is significant at the 0.05 level. One-way ANOVA with a Tukey-HSD post hoc test was applied to see the statically significant difference.

**Table 4:** Association of PON-1 and SRB-1 level with demographic characteristics.

		Cases					Controls				
		*n* (%)	PON-1 Mean ± SD	*p* Value	SRB-1 Mean ± SD	*p* Value	*n* (%)	PON-1 Mean ± SD	*p* Value	SRB-1 Mean ± SD	*p* Value
Tobacco use	Yes	46 (66.67)	32.76 ± 15.85	0.932	12.37 ± 3.26	0.942	13 (56.5)	61.96 ± 11.73	0.584	15.31 ± 2.82	0.654
	No	23 (33.3)	32.43 ± 12.05		12.73 ± 3.48		10 (43.5)	58.81 ± 13.42		16.40 ± 2.45	
Alcohol use	Yes	8 (11.6)	27.14 ± 10.56	0.26	13.47 ± 3.53	0.823	2 (8.7)	59.75 ± 2.25	0.513	14.19 ± 0.09	0.587
	No	61 (88.4)	33.37 ± 14.86		12.35 ± 3.26		21 (91.3)	60.77 ± 12.75		15.91 ± 2.81	
Family history	Yes	4 (5.8)	32.29 ± 14.47	0.419	12.55 ± 3.39	0.523	2 (8.7)	74.75 ± 3.95	0.105	14.99 ± 1.69	0.432
	No	65 (94.2)	38.45 ± 14.91		11.36 ± 1.15		21 (91.3)	59.27 ± 12.24		15.83 ± 2.80	
Obesity	Yes	10 (14.5)	36.86 ± 10.81	0.330	12.30 ± 2.56	0.895	5 (21.7)	66.47 ± 12.05	0.329	17.30 ± 2.70	0.422
	No	59 (85.5)	31.93 ± 14.99		12.51 ± 3.42		18 (78.3)	59.38 ± 12.29		15.41 ± 2.61	
Hypertension	Yes	30 (43.5)	38.97 ± 16.08	0.002	12.73 ± 3.37	0.622	5 (21.7)	57.52 ± 13.27	0.545	18.49 ± 1.48	0.983
	No	39 (56.5)	28.34 ± 11.33		12.32 ± 3.29		18 (78.3)	61.60 ± 12.17		14.95 ± 2.48	

**Table 5:** Diagnostic value of PON-1 and SRB-1 in differentiation of CAD from controls.

Marker	Cutoff Value	AUC	*p* Value	Sensitivity (95% CI)	Specificity (95% CI)
PON-1					
Case vs. Control	<39.10	0.93	<0.0001	75.36 (64.04–84.01)	100.0 (85.13–100.0)
Category 1 vs. Control	<44.90	0.86	<0.0001	73.91 53.53–87.45	90.91 (72.19–98.38)
Category 2 vs. Control	<38.15	0.94	<0.0001	73.91 (53.53–87.45)	100.0 (85.13–100.0)
Category 3 vs. Control	<39.10	0.99	<0.0001	95.65 (79.01–99.78)	100.0 (85.13–100.0)
SRB-1					
Case vs. Control	<13.63	0.75	0.0005	57.97 46.21–68.89	72.73 51.85–86.85
Category 1 vs. Control	<14.94	0.62	0.112	60.87 40.79–77.84	54.55 34.66–73.08
Category 2 vs. Control	<13.63	0.72	0.008	56.52 (36.81–74.37)	72.73 (51.85–86.85)
Category 3 vs. Control	<12.75	0.88	<0.001	78.26 (58.10–90.34)	90.91 (72.19–98.38)

## Data Availability

The data presented in this study are available upon request from the corresponding author.

## References

[1] Rao G.H. (2018). Number one killer: Vascular disease. Ann. Clin. Diabetes Endocrinol..

[2] Prabhakaran D., Jeemon P., Roy A. (2016). Cardiovascular diseases in India: Current epidemiology and future directions. Circulation.

[3] Kratzer A., Giral H., Landmesser U. (2014). High-density lipoproteins as modulators of endothelial cell functions: Alterations in patients with coronary artery disease. Cardiovasc. Res..

[4] Kontush A., Chapman M.J. (2006). Functionally defective high-density lipoprotein: A novel therapeutic target at the crossroads of dyslipidemia, inflammation, and atherosclerosis. Pharmacol. Rev..

[5] Gong M., Wilson M., Kelly T., Su W., Dressman J., Kincer J., Matveev S.V., Guo L., Guerin T., Li X.A. (2003). HDL-associated estradiol stimulates endothelial NO synthase and vasodilation in an SR-BI–dependent manner. J. Clin. Investig..

[6] Mackness B., Durrington P.N., Mackness M.I. (2002). Paraoxonase gene family and coronary heart disease. Curr. Opin. Lipidol..

[7] Durrington P.N., Mackness B., Mackness M.I. (2001). Paraoxonase and atherosclerosis. Arter. Thromb. Vasc. Biol..

[8] McElveen J., Mackness M.I., Colley C.M., Peard T., Warner S., Walker C.H. (1986). Distribution of paraoxon hydrolytic activity in the serum of patients after myocardial infarction. Clin. Chem..

[9] Patra S.K., Singh K., Singh R. (2013). Paraoxonase-1: A better atherosclerotic risk predictor than HDL in type 2 diabetes mellitus. Diabetes Metab. Syndr. Clin. Res. Rev..

[10] Esparragón F.R., Trujillo Y.H., Reyes A.M., Ortega E.H., Medina A., Pérez J.C. (2006). Concerning the significance of paraoxonase-1 and SRB-1 genes in atherosclerosis. Rev. Española Cardiol..

[11] McCarthy J.J., Lehner T., Reeves C., Moliterno D.J., Newby L.K., Rogers W.J., Topol E.J. (2003). Association of genetic variants in the HDL receptor, SRB-1, with abnormal lipids in women with coronary artery disease. J. Med. Genet..

[12] Saddar S., Carriere V., Lee W.R., Tanigaki K., Yuhanna I.S., Parathath S., Morel E., Warrier M., Sawyer J.K., Gerard R.D. (2013). Scavenger receptor class B type I is a plasma membrane cholesterol sensor. Circ. Res..

[13] Zhang Y., Da Silva J.R., Reilly M., Billheimer J.T., Rothblat G.H., Rader D.J. (2005). Hepatic expression of scavenger receptor class B type I (SR-BI) is a positive regulator of macrophage reverse cholesterol transport in vivo. J. Clin. Investig..

[14] King S.B., Smith S.C., Hirshfeld J.W., Jacobs A.K., Morrison D.A., Williams D.O., Feldman T.E., Kern M.J., O’Neill W.W., Schaff H.V. (2008). 2007 Focused Update of the ACC/AHA/SCAI 2005 Guideline Update for Percutaneous Coronary Intervention: A Report of the American College of Cardiology/American Heart Association Task Force on Practice Guidelines: 2007 Writing Group to Review New Evidence and Update the ACC/AHA/SCAI 2005 Guideline Update for Percutaneous Coronary Intervention, Writing on Behalf of the 2005 Writing Committee. Circulation.

[15] Joshi P., Islam S., Pais P., Reddy S., Dorairaj P., Kazmi K., Pandey M.R., Haque S., Mendis S., Rangarajan S. (2007). Risk factors for early myocardial infarction in South Asians compared with individuals in other countries. JAMA.

[16] Geldsetzer P., Manne-Goehler J., Theilmann M., Davies J.I., Awasthi A., Danaei G., Gaziano T.A., Vollmer S., Jaacks L.M., Baernighausen T. (2018). Geographic and sociodemographic variation of cardiovascular disease risk in India: A cross-sectional study of 797,540 adults. PLoS Med..

[17] Sun T., Hu J., Yin Z., Xu Z., Zhang L., Fan L., Zhuo Y., Wang C. (2017). Low serum paraoxonase-1 activity levels predict coronary artery disease severity. Oncotarget.

[18] Iaccarino G., Ciccarelli M., Sorriento D., Galasso G., Campanile A., Santulli G., Cipolletta E., Cerullo V., Cimini V., Altobelli G.G. (2005). Ischemic neoangiogenesis enhanced by β2-adrenergic receptor overexpression: A novel role for the endothelial adrenergic system. Circ. Res..

[19] Gupta N., Singh S., Maturu V.N., Sharma Y.P., Gill K.D. (2011). Paraoxonase-1 (PON-1) polymorphisms, haplotypes and activity in predicting cad risk in North-West Indian Punjabis. PLoS ONE.

[20] Litvinov D., Mahini H., Garelnabi M. (2012). Antioxidant and anti-inflammatory role of paraoxonase-1: Implication in arteriosclerosis diseases. N. Am. J. Med. Sci..

[21] Fortunato G., Rubba P., Panico S., Trono D., Tinto N., Mazzaccara C., De Michele M., Iannuzzi A., Vitale D.F., Salvatore F. (2003). A paraoxonase gene polymorphism, PON-1 (55), as an independent risk factor for increased carotid intima-media thickness in middle-aged women. Atherosclerosis.

[22] Coombes R.H., Crow J.A., Dail M., Chambers H.W., Wills R.W., Chambers J.E., Bertolet B.D. (2011). for CARe. Relationship of Human Paraoxonase-1 (PON-1) Serum Activity and Genotype with Atherosclerosis in Individuals from the Deep South. Pharmacogenet. Genom..

[23] Bhaskar S., Ganesan M., Chandak G.R., Mani R., Idris M.M., Khaja N., Gulla S., Kumar U., Movva S., Vattam K.K. (2011). Association of PON-1 and APOA 5 gene polymorphisms in a cohort of Indian patients having coronary artery disease with and without type 2 diabetes. Genet. Test. Mol. Biomark..

[24] Efrat M., Aviram M. (2008). Macrophage paraoxonase-1 (PON-1) binding sites. Biochem. Biophys. Res. Commun..

[25] Ahmed Z., Babaei S., Maguire G.F., Draganov D., Kuksis A., La Du B.N., Connelly P.W. (2003). Paraoxonase-1 reduces monocyte chemotaxis and adhesion to endothelial cells due to oxidation of palmitoyl, linoleoyl glycerophosphorylcholine. Cardiovasc. Res..

[26] Chistiakov D.A., Melnichenko A.A., Orekhov A.N., Bobryshev Y.V. (2017). Paraoxonase and atherosclerosis-related cardiovascular diseases. Biochimie.

[27] Mackness M.I., Arrol S., Mackness B., Durrington P.N. (1997). Alloenzymes of paraoxonase and effectiveness of high-density lipoproteins in protecting low-density lipoprotein against lipid peroxidation. Lancet.

[28] Out R., Hoekstra M., Spijkers J.A., Kruijt J.K., van Eck M., Bos I.S., Twisk J., Van Berkel T.J. (2004). Scavenger receptor class B type I is solely responsible for the selective uptake of cholesteryl esters from HDL by the liver and the adrenals in mice. J. Lipid Res..

[29] Ji Y., Wang N., Ramakrishnan R., Sehayek E., Huszar D., Breslow J.L., Tall A.R. (1999). Hepatic scavenger receptor BI promotes rapid clearance of high-density lipoprotein free cholesterol and its transport into bile. J. Biol. Chem..

[30] Varban M.L., Rinninger F., Wang N., Fairchild-Huntress V., Dunmore J.H., Fang Q., Gosselin M.L., Dixon K.L., Deeds J.D., Acton S.L. (1998). Targeted mutation reveals a central role for SR-BI in hepatic selective uptake of high-density lipoprotein cholesterol. Proc. Natl. Acad. Sci. USA.

[31] Rigotti A., Trigatti B.L., Penman M., Rayburn H., Herz J., Krieger M. (1997). A targeted mutation in the murine gene encoding the high-density lipoprotein (HDL) receptor scavenger receptor class B type I reveals its key role in HDL metabolism. Proc. Natl. Acad. Sci. USA.

[32] Braun A., Trigatti B.L., Post M.J., Sato K., Simons M., Edelberg J.M., Rosenberg R.D., Schrenzel M., Krieger M. (2002). Loss of SR-BI expression leads to the early onset of occlusive atherosclerotic coronary artery disease, spontaneous myocardial infarctions, severe cardiac dysfunction, and premature death in apolipoprotein E–deficient mice. Circ. Res..

[33] Covey S.D., Krieger M., Wang W., Penman M., Trigatti B.L. (2003). Scavenger receptor class B type I–mediated protection against atherosclerosis in LDL receptor–negative mice involve its expression in bone marrow–derived cells. Arter. Thromb. Vasc. Biol..

[34] Huszar D., Varban M.L., Rinninger F., Feeley R., Arai T., Fairchild-Huntress V., Donovan M.J., Tall A.R. (2000). Increased LDL Cholesterol and Atherosclerosis in LDL Receptor–Deficient Mice with Attenuated Expression of Scavenger Receptor B1. Arter. Thromb. Vasc. Biol..

[35] Wang N., Arai T., Ji Y., Rinninger F., Tall A.R. (1998). Liver-specific overexpression of scavenger receptor BI decreases levels of very low-density lipoprotein ApoB, low-density lipoprotein ApoB, and high-density lipoprotein in transgenic mice. J. Biol. Chem..

[36] Ueda Y., Royer L., Gong E., Zhang J., Cooper P.N., Francone O., Rubin E.M. (1999). Lower plasma levels and accelerated clearance of high-density lipoprotein (HDL) and non-HDL cholesterol in scavenger receptor class B type I transgenic mice. J. Biol. Chem..

[37] Webb N.R., de Beer M.C., Yu J., Kindy M.S., Daugherty A., van der Westhuyzen D.R., de Beer F.C. (2002). Overexpression of SR-BI by adenoviral vector promotes clearance of apoA-I, but not apoB, in human apoB transgenic mice. J. Lipid Res..

[38] Kozarsky K.F., Donahee M.H., Rigotti A., Iqbal S.N., Edelman E.R., Krieger M. (1997). Overexpression of the HDL receptor SR-BI alters plasma HDL and bile cholesterol levels. Nature.

[39] Kozarsky K.F., Donahee M.H., Glick J.M., Krieger M., Rader D.J. (2000). Gene transfer and hepatic overexpression of the HDL receptor SR-BI reduces atherosclerosis in the cholesterol-fed LDL receptor–deficient mouse. Arter. Thromb. Vasc. Biol..

[40] Zanoni P., Khetarpal S.A., Larach D.B., Hancock-Cerutti W.F., Millar J.S., Cuchel M., DerOhannessian S., Kontush A., Surendran P., Saleheen D. (2016). Rare variant in scavenger receptor BI raises HDL cholesterol and increases risk of coronary heart disease. Science.

[41] Koenig S.N., Sucharski H.C., Jose E.M., Dudley E.K., Madiai F., Cavus O., Argall A.D., Williams J.L., Murphy N.P., Keith C.B. (2021). Inherited variants in SCARB1 cause severe early onset coronary artery disease. Circ. Res..

[42] Huang L., Chambliss K.L., Gao X., Yuhanna I.S., Behling-Kelly E., Bergaya S., Ahmed M., Michaely P., Luby-Phelps K., Darehshouri A. (2019). SRB-1 drives endothelial cell LDL transcytosis via DOCK4 to promote atherosclerosis. Nature.

[43] Wang M., Li L., Liu R., Song Y., Zhang X., Niu W., Kumar A.K., Guo Z., Hu Z. (2018). Obesity-induced overexpression of miRNA-24 regulates cholesterol uptake and lipid metabolism by targeting SRB-1. Gene.

[44] Shen W.J., Azhar S., Kraemer F.B. (2018). SRB-1: A unique multifunctional receptor for cholesterol influx and efflux. Annu. Rev. Physiol..

[45] Jiang C. The Role of SRB-1 in Lipid Metabolism and Inflammation in 3T3-L1 Adipocytes. https://opencommons.uconn.edu/usp_projects/35.

[46] Perrone M.A., Donatucci B., Salvati A., Gualtieri P., De Lorenzo A., Romeo F., Bernardini S. (2019). Inflammation, oxidative stress and gene expression: The postprandial approach in professional soccer players to reduce the risk of muscle injuries and early atherosclerosis. Med. Sport.

[47] López-Candales A., Sawalha K. (2023). Improving diagnostic assessments in the ever-changing landscape of atherosclerosis. J. Cardiovasc. Med..

[48] Perrone M.A., Gualtieri P., Gratteri S., Ali W., Sergi D., Muscoli S., Cammarano A., Bernardini S., Di Renzo L., Romeo F. (2019). Effects of postprandial hydroxytyrosol and derivates on oxidation of LDL, cardiometabolic state and gene expression: A nutrigenomic approach for cardiovascular prevention. J. Cardiovasc. Med..

[49] Cimmino G., Muscoli S., De Rosa S., Cesaro A., Perrone M.A., Selvaggio S., Selvaggio G., Aimo A., Pedrinelli R., Mercuro G. (2023). Evolving concepts in the pathophysiology of atherosclerosis: From endothelial dysfunction to thrombus formation through multiple shades of inflammation. J. Cardiovasc. Med..

